# *jmodeltest*.org: selection of nucleotide substitution models on the cloud

**DOI:** 10.1093/bioinformatics/btu032

**Published:** 2014-01-21

**Authors:** Jose Manuel Santorum, Diego Darriba, Guillermo L. Taboada, David Posada

**Affiliations:** ^1^Department of Electronics and Systems, University of A Coruña, 15071 A Coruña and ^2^Department of Biochemistry, Genetics and Immunology, University of Vigo, 36201 Vigo, Spain

## Abstract

**Summary:** The selection of models of nucleotide substitution is one of the major steps of modern phylogenetic analysis. Different tools exist to accomplish this task, among which jModelTest 2 (jMT2) is one of the most popular. Still, to deal with large DNA alignments with hundreds or thousands of loci, users of jMT2 need to have access to High Performance Computing clusters, including installation and configuration capabilities, conditions not always met. Here we present *jmodeltest*.org, a novel web server for the transparent execution of jMT2 across different platforms and for a wide range of users. Its main benefit is straightforward execution, avoiding any configuration/execution issues, and reducing significantly in most cases the time required to complete the analysis.

**Availability and implementation:** *jmodeltest*.org is accessible using modern browsers, such as Firefox, Chrome, Opera, Safari and IE from http://jmodeltest.org. User registration is not mandatory, but users wanting to have additional functionalities, like access to previous analyses, have the possibility of opening a user account.

**Contact:** info@jmodeltest.org

## 1 INTRODUCTION

The statistical selection of best-fit models of nucleotide substitution is relevant for the phylogenetic analysis of DNA sequence alignments (Sullivan and Joyce, 2005). With the advent of next-generation sequencing (NGS) technologies, many researches are moving from phylogenetics to phylogenomics, in which large sequence alignments typically include hundreds or thousands of loci. Phylogenetic resources, therefore, need to be adapted to a high-performance computing paradigm so as to allow demanding analyses. To keep up with the increasing availability of genome-wide data, jModelTest 2 (jMT2) (Darriba *et al.*, 2012) was recently developed to profit from technical optimizations and parallel computing. jMT2 uses PhyML (Guindon and Gascuel, 2003) to obtain maximum likelihood estimates of model parameters, and implements different statistical criteria for model selection including hierarchical and dynamical likelihood ratio tests, Akaike’s and Bayesian information criteria (AIC and BIC) and a performance-based decision theory method (Posada and Buckley, 2004). jMT2 can take advantage of high-performance computing (HPC) environments, such as supercomputers and clusters. However, execution in HPC environments is not trivial: (i) installing, configuring and optimizing parallel software are generally cumbersome for non-HPC experts, (ii) access to HPC resources generally implies long waiting times or at least significant variability in the response time and (iii) it is difficult to estimate in advance the computational resources needed.

To overcome these limitations, we introduce *jmodeltest*.org, a web service for executing jMT2 transparently on HPC infrastructures. *jmodeltest*.org can distribute jMT2 jobs across multiple public or private clouds, such as Amazon Web Services (AWS) EC2, adopting optimal HPC configurations. *jmodeltest*.org considers the available resources at each site to minimize execution times and scales the resources up and down depending on the workload. Such an ‘easy’ access to HPC resources will allow users to focus more on their research rather than on secondary tasks like resource provision, installation, configuration, execution and optimization of parallel environments.

## 2 IMPLEMENTATION

*jmodeltest*.org has been implemented as a web interface for jMT2, plus a task manager. The web interface captures input data and parameters, whereas the task manager divides jMT2 jobs in different subtasks, one per substitution model. *jmodeltest*.org looks for infrastructures, which are ready to execute these subtasks immediately. Currently, *jmodeltest*.org jobs will run in private clouds at the University of Vigo and University of A Coruña, and occasionally at the Galicia Supercomputing Center (CESGA) and Amazon WS EC2 public clouds. When the server workload exceeds the available capacity of the private clouds, resources are requested from the public clouds. The technologies behind *jmodeltest*.org are Tomcat for the web interface, MySQL for handling subtasks, DRMAA for executing tasks on remote servers and *StarCluster* (http://star.mit.edu/cluster/) for managing Amazon WS EC2 resources.

Because the tasks are split, *jmodeltest*.org is able to start large analyses without having yet assigned computational resources for the whole job. Subtasks are sent to the different computational resources through Distributed Resource Management Application API (DRMAA), a high-level Open Grid Forum API specification for the submission and control of jobs to a Distributed Resource Management (DRM) system, such as a Cluster or a Grid computing infrastructure. As the job manager is not aware of the resources required to run a particular task, it will start submitting 1h jobs with 1 GB of memory. This way, cloud schedulers will allocate resources faster. In case these initial requests are not enough, subsequent submissions will double either the time and/or the amount of memory. To save resources, *jmodeltest*.org implements a check-pointing mechanism using Distributed Multi-Threaded Check-Pointing (DMTCP).

Furthermore, users will be able use their own computational resources when running *jmodeltest*.org. The only requirement is that these machines have a resource manager (i.e. SGE, Torque, SLURM) with proper user permissions. After this, the user just needs to register this resource in *jmodeltest*.org. Only the user who registered the resource will be able to execute *jmodeltest*.org jobs on it. Communications with the added resource are secured through a public RSA key 1024 bits. Finally, we are working on a new feature that will allow users to request exclusive access to prepaid AWS EC2 resources for accelerating the jobs.

## 3 FUNCIONALITY

*jmodeltest*.org was designed to be completely transparent to the user, who does not need to install, configure or update anything, nor specifying the resources needed in a shared resources infrastructure, like the number of cores or user permissions. *jmodeltest*.org is accessible through any web browser. Users can login anonymously or register. If the login is anonymous, analyses are executed within a web session, until the browser is closed or there is a long inactivity period, losing any resulting jobs. When access occurs through a user account, job settings and results are kept in the server, and registered users can recover these at any time. This can be particularly interesting when analysing large datasets, avoiding accidental interruptions. The user account can be accessed multiple times and from multiple devices. Moreover, *jmodeltest*.org helps users to monitor their jobs, displaying information about their current state (‘initializing’, ‘running’, ‘done’) and resources consumed (CPU time). Once the job is completed, the user can output, view, download or delete the results. By default, *jmodeltest*.org limits the CPU time granted per user to (currently) 500 CPU hours. The web service includes documentation, example files, support tickets and a FAQ section.

## 4 PERFORMANCE

**Fig. 1.**
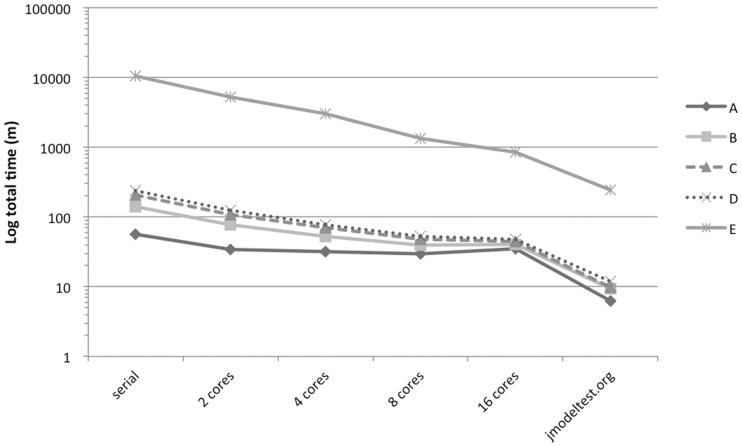
Execution times for five datasets (alignments A–E, with 35, 43, 9, 44 and 246 sequences and 392, 492, 14 403, 561 and 4465 sites, respectively), using the serial and parallel versions of jModelTest2, and *jmodeltest*.org

For benchmarking, we submitted five representative datasets to *jmodeltest*.org. We recorded the time to complete the likelihood calculations, by far the most intensive task, for 88 models using default settings, for (i) the serial version of jMT2 running in a single core, (ii) the parallel version of jMT2 running on 2, 4, 8 and 16 cores on a shared resource and (iii) *jmodeltest*.org running on backend private clouds and public cloud providers (CESGA and AWS), without waiting for resources and virtually running all tasks in parallel. Figure 1 presents the resulting execution times, taking into account both queuing time and runtime. The queuing time increased with the number of cores requested, reducing significantly the benefits of using the parallel version on a shared resource. Here *jmodeltest*.org performed best, as it has multiple resources and can virtually run all the tasks in parallel.
